# Displacement Effects of Conservation Grazing on Red Deer (*Cervus elaphus*) Spatial Behaviour

**DOI:** 10.1007/s00267-022-01697-6

**Published:** 2022-08-22

**Authors:** Fabio Weiss, Frank Uwe Michler, Benjamin Gillich, Jörg Tillmann, Simone Ciuti, Marco Heurich, Siegfried Rieger

**Affiliations:** 1 Biosphere Reserves Institute, Eberswalde University for Sustainable Development, Eberswalde, Germany; 2 Department Wildlife Ecology and Management, University of Freiburg, Breisgau, Germany; 3 Department of Wildlife Biology, Wildlife Management and Hunting Practice, Eberswalde University for Sustainable Development, Eberswalde, Germany; 4 Institute of Zoology, University of Hamburg, Hamburg, Germany; 5 DBU Natural Heritage GmbH, Osnabrück, Germany; 6 Laboratory of Wildlife Ecology and Behaviour, SBES, University College Dublin, Dublin, Ireland; 7 Department of Conservation and Research, Bavarian Forest National Park, Bavaria, Germany; 8 Institute for Forest and Wildlife Management, Inland Norway University for Applied Science, Hamar, Norway

**Keywords:** Targeted grazing, Heathland conservation, Dry grassland, Wildlife, Habitat selection, Resource selection functions

## Abstract

Conservation grazing uses semi-feral or domesticated herbivores to limit encroachment in open areas and to promote biodiversity. However, we are still unaware of its effects on wild herbivores. This study investigates the influence of herded sheep and goats on red deer (*Cervus elaphus*) spatial behavior by testing three a-priori hypotheses: (i) red deer are expected to avoid areas used by livestock, as well as adjacent areas, when livestock are present, albeit (ii) red deer increase the use of these areas when sheep and goats are temporarily absent and (iii) there is a time-lagged disruption in red deer spatial behavior when conservation grazing practice ends. Using GPS-telemetry data on red deer from a German heathland area, we modelled their use of areas grazed by sheep and goats, using mixed-effect logistic regression. Additionally, we developed seasonal resource selection functions (use-availability design) to depict habitat selection by red deer before, during, and after conservation grazing. Red deer used areas less during conservation grazing throughout all times of the day and there was no compensatory use during nighttime. This effect mostly persisted within 21 days after conservation grazing. Effects on habitat selection of red deer were detectable up to 3000 meters away from the conservation grazing sites, with no signs of either habituation or adaption. For the first time, we demonstrate that conservation grazing can affect the spatio-temporal behavior of wild herbivores. Our findings are relevant for optimizing landscape and wildlife management when conservation grazing is used in areas where wild herbivores are present.

## Introduction

Semi-natural open areas such as grasslands, heathlands or wetlands often act as important refuges for rare, highly specialized plant and animal species (Luoto et al. 2003; Warren et al. 2010; Benthien et al. 2018; Riesch et al. 2020) as a result of natural open areas being degraded or lost to land-use change (Carbutt et al. 2017). These ecosystems and their associated species, however, are increasingly under pressure due to the natural succession towards closed forests (Pakeman et al. 2003; Buchholz et al. 2013; Koch et al. 2015). Open areas with a history of anthropogenic use (e.g., military training sites or extensive pastures) particularly experience encroachment by shrubs or trees often resulting in a loss of biodiversity after human activities cease or decrease (Luoto et al. 2003; Warren et al. 2010).

In the past decades, targeted low-intensity grazing with semi-feral or domesticated livestock—also called conservation grazing (sensu Bailey et al. 2019)—has gained popularity as a tool for maintaining or restoring semi-natural open landscapes by counteracting natural succession (e.g., van Wieren 1995; Dostálek and Frantík 2008; Jauregui et al. 2009). Through mechanisms such as browsing, trampling, defecation, and seed dispersal, conservation grazing can additionally increase plant species diversity, structural diversity, and species turnover (Bakker et al. 1983; Dostálek and Frantík 2008; Benthien et al. 2018; Riesch et al. 2020). Moreover, low-intensity grazing regimes have also been found to enhance overall faunistic biodiversity (van Wieren and Bakker 2008; but see Reading and Jofré, 2015). For example, beneficial effects have been described for species richness and density of birds (Zalba and Cozzani 2004) or species richness and turnover of spiders (Dennis et al. 2015). Further, low-intensity grazing can help to maintain unique species assemblages of ecosystems sensitive to shrub- or tree-encroachment, for instance in carabid beetles (Schirmel et al. 2015). Therefore, conservation grazing has become an important management tool in nature conservation when it comes to maintaining or restoring open landscapes such as grass- and heathland (Newton et al. 2009). However, it is not currently clear to what extent semi-feral or domesticated herbivores interact with and influence wild ungulate herbivores, as research investigating the ecological consequences of conservation grazing has mainly focused on impacts on vegetation (Gallet and Roze 2001; Jauregui et al. 2009; Benthien et al. 2018) and smaller animal species (van Wieren and Bakker 2008; Schirmel et al. 2015; Schwerk et al. 2021). But also wild herbivores have the potential of maintaining open landscapes and enhance biodiversity (van Wieren and Bakker 2008). Red deer (*Cervus elaphus*) are large ungulates that display grazing-type feeding behavior and often utilize open areas such as grass- and heathland for foraging (Wolff and Horn 2003; Godvik et al. 2009; Meißner et al. 2012). Riesch et al. (2019) found in a field experiment that the quantity of biomass removed by wild red deer in semi-natural grass- and heathland is comparable to that theoretically achieved by livestock at stocking rates recommended for conservation grazing. Furthermore, a study by Hester and Baillie (1998) on enclosed heathland plots showed that the grazing impact of red deer can even exceed that of sheep, even when red deer were present at lower densities than sheep (12 vs. 8 animals h^−1^). Moreover, Riesch et al. (2020) observed an increase in vegetation height and the encroachment of woody vegetation following the exclusion of red deer. In the case of conservation grazing the similar use of resources might lead to competition between domesticated and wild herbivores such as red deer.

In the anthropogenic landscapes of central Europe, large semi-natural open areas nestling within forested areas often represent important refuges for wildlife such as red deer, which typically use these tracts of land for foraging (van Wieren and Bakker 2008) and, to a lesser (but equally important) extent, for mating (Meißner et al. 2012). These areas are usually less exposed to human activities (e.g., agriculture, recreational activities, traffic) and might enable red deer to adopt less disturbed activity patterns: Red deer are often described to have crepuscular activity rhythms showing peaks of activity around sunrise and sunset (e.g., Clutton-Brock et al. 1982; Godvik et al. 2009; Ensing et al. 2014). However, in absence of disturbance, these peaks seem to be less pronounced (Kamler et al., 2007), and diurnal activity increases (Ensing et al. 2014). Red deer are known to be sensitive to disturbances (Edge and Marcum 1985; Czech 1991; Sibbald et al. 2011), especially in open areas, as these lack potential cover in which to hide (Jayakody et al. 2008; Stankowich 2008). They are also reportedly sensitive to rapid movements and noise, either of which usually induces a flight response (Frid and Dill 2002), and it was found that disturbances can still affect their spatial behavior at large distances away from the actual source of disturbance (Edge and Marcum 1985). Several studies have reported that free-ranging red deer avoid areas used by cattle (Stewart et al. 2002; Coe et al. 2004; Pruvot et al. 2014), whilst a study by Hester et al. (1999) found that red deer and sheep are weakly affected by each other’s presence or absence in the extensive heath moorland of north Scotland. Nevertheless, to the best of our knowledge, no research has investigated how free-ranging red deer react to conservation grazing, particularly in the context of central European dry grass- or heathland. Herding is a common practice in conservation grazing (Bailey et al. 2019), which avoids fencing and therefore also a direct exclusion of larger wildlife. However, shepherds and their dogs may be perceived as predators and possibly trigger avoidance behavior in red deer. A number of studies reports that they avoid areas with a high wolf predation risk and withdraw to more sheltered areas (Wolff and Horn 2003; Creel et al. 2005; Hernández and Laundré 2005) and a study by van Beeck Calkoen et al. (2022) showed that risk effects of human activities can even outweigh those of predators.

On the other hand, there is evidence that red deer are able to adapt to predation risk by altering their temporal use of certain areas rather than avoiding them completely (risky times hypothesis vs. risky places hypothesis, Creel et al. 2008). They have also been found to habituate or adapt to human activities, as well as spatially and temporally evade different kinds of disturbances (Thompson and Henderson 1998; Sibbald et al. 2011; Westekemper et al. 2018). For example, a study by Edge and Marcum (1985) observed that they avoid areas during ongoing logging operations and return on weekends when logging is paused. Similarly, they have been found to avoid areas close to hiking trails during daytime, when they are more frequented while returning at night (Marion et al. 2021). This ability to adapt to disturbances seems to primarily occur whenever these follow a regular pattern, but it is not evident when they are not predictable (Knight 1980; Westekemper et al. 2018). Despite this ability to adapt to disturbances, several studies acknowledge red deer site-fidelity (Switzer 1993) or spatial memory (Fagan et al. 2013) as important factors in habitat selection, meaning that they show a tendency to use territories with which they are familiar (Wolf et al. 2009; Gautestad et al. 2013). Conversely, this could mean that once they have adapted their spatial behavior to a predictable and long-lasting disturbance, they will continue to display this altered behavior in excess of the actual disturbance (e.g., Sibbald et al. 2011). Additionally, persistent scents, especially those of dogs, could negatively affect the attractiveness of these areas for extended periods (Chabot et al. 1996; Elmeros et al. 2011). To date, the scientific literature has insufficiently covered conservation grazing and its direct and indirect influences on mammalian wildlife. Particularly, very little information is available regarding herded sheep or goats and wild red deer (but see Hester et al. 1999).

In this study, we investigate the effects of conservation grazing with herded sheep and goats on the spatio-temporal behavior of wild red deer in dry heathland. In particular, we are interested in displacement effects, the temporal scale of such potentially time-lagged effects, and any signs of either adaption or habituation to conservation grazing. Specifically, we hypothesize that (i) red deer are expected to avoid areas used by livestock, as well as adjacent areas, when livestock are present, albeit (ii) red deer increase the use of these areas when sheep and goats are temporarily absent and (iii) there is a time-lagged disruption in red deer spatial behavior when conservation grazing practice ends. A potential displacement of red deer by targeted sheep-grazing—either due to resource competition or direct disturbance—would be highly relevant for both, wildlife management and conservation of semi-natural open areas.

## Methods

### Study Area

**Fig. 1:**
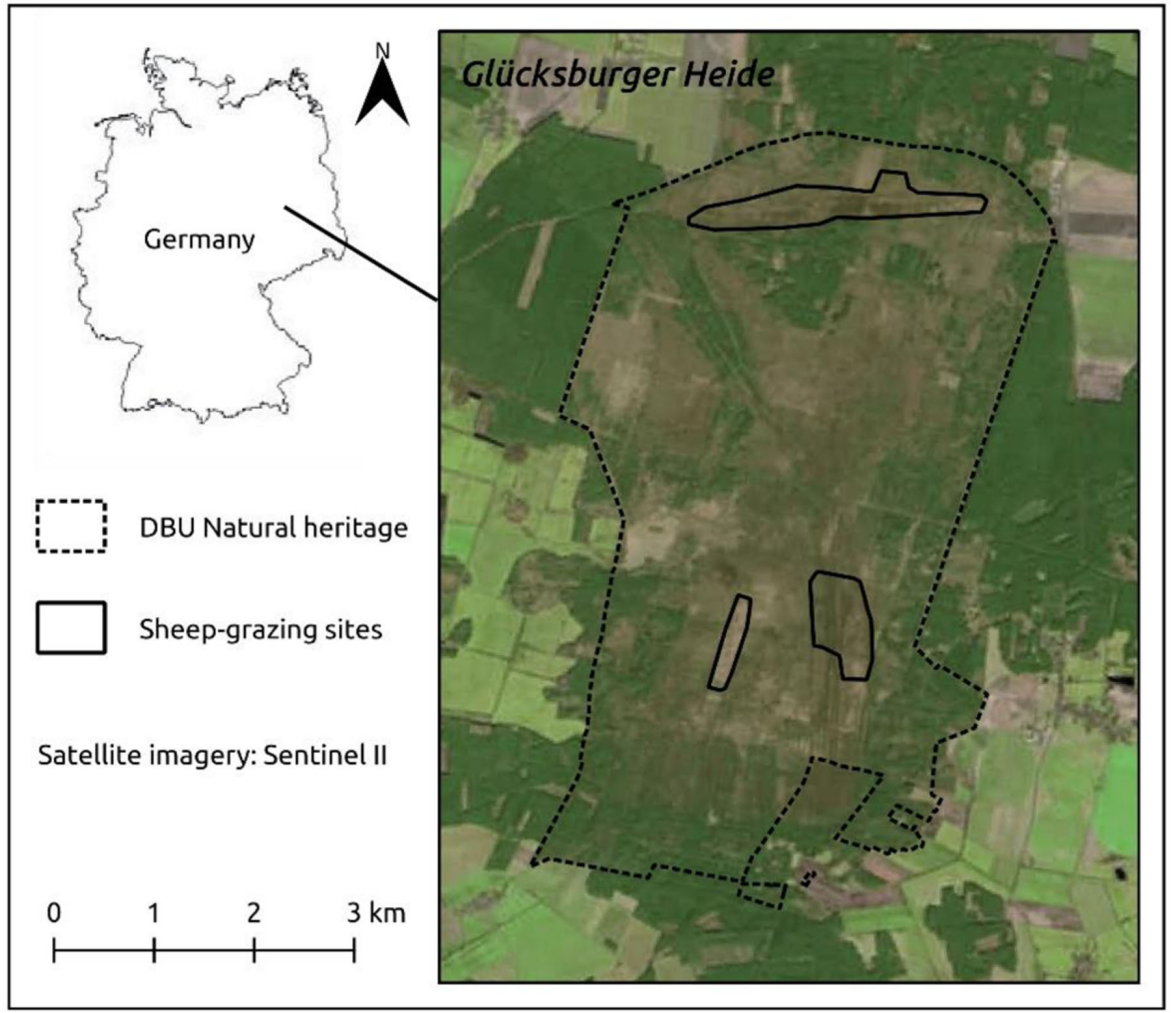
Map of the Glücksburger Heide study area in Saxony-Anhalt; the three conservation grazing sites are indicated by black lines. Borders of the DBU-managed Natural Heritage area are marked with black-dotted lines

We conducted our research at Glücksburger Heide, a 7000-hectare former military training site located in Saxony-Anhalt, Germany (WGS84: 51.880556, 12.983361) (Fig.1). The 2600-hectare core area was declared a dedicated National Natural Heritage site in 2009, and it is owned and managed by the German Natural Heritage GmbH (DBU Naturerbe GmbH). It is also a declared NATURA 2000 site (“Glücksburger Heide” DE 4143-401) under the EU habitats directive and the EU birds directive. At its centre, the site features large areas of dry heathland with common heather (Calluna vulgaris) and, to a lesser extent, xeric grassland with grey hair-grass (*Corynephorus canescens*). These open areas are surrounded by forest stands of different age and species composition, mainly consisting of pine (*Pinus sylvestris*) and birch (*Betula pendula*). Glücksburger Heide itself is situated in an agricultural landscape. Red deer are very abundant in the study area, with an estimated density of 11.6 individuals per km². Hunting activities aimed at red deer, wild boar (*Sus scrofa*) and roe deer (*Capreolus capreolus*) include two driven hunts^1^ in the winter and, to a lesser degree, interval hide hunting in May and the period from September to December. Officially, public access is only permitted along one road crossing the center of Glücksburger Heide in east-west direction (Fig.1) and on a small number of marked walking routes. Thus, recreational use of the area can be assumed to be very limited (Gillich et al. 2021).

### Conservation Grazing and Red Deer Data

In 2016, managing authorities deployed herded sheep (Heidschnucke and Romanov sheep) and goats (German Edelziege) for conservation grazing on different successional grass- and heathland areas within the study area. From 2016 to 2018, conservation grazing took place at three distinct sites comprising 62, 22, and 102 hectares, respectively (Fig. 1). The grazing intervals took place at different times of the year and varied in length (Table SI.1, supplementary information). In 2016, sheep and goats grazed in December. In 2017 and 2018, conservation grazing started in June and lasted until September and October, respectively. The sheep first grazed at either the most northern site or two southern sites for a consecutive period, before then being moved to the respective other site for the following period. Sheep and goats grazed together as one single flock. Both livestock species are commonly used for conservation grazing, while a combination of both species is also not uncommon (Marchetto et al. 2021). From 2016 to 2018, this flock consisted of 180 to 198 sheep and 13 to 24 goats, yielding a total number of between 204 and 212 animals (for reasons of simplicity both, sheep and goats, are simply referred to as sheep in the following). The sheep were escorted by one shepherd and one to two sheepdogs. The sheep, shepherd, and dog(s) only used the grazing sites during the day, with the flock being moved to pens at night. The shepherd and their dog(s) did not spend the night in the area. During grazing activities in 2017, one goat was equipped with a GSM-GPS collar (Vectronic Aerospace, Pro Light), which recorded its position every two hours. We used GPS-relocation of this goat to define the limits of the grazing sites, by using the minimum convex polygons function in QGIS (QGIS Development Team 2018). Additional conservation measures at the three distinct sides included the manual removal of tree saplings and the mowing of heather.

Between 2014 and 2018, a total of 25 red deer (14 hinds, eleven stags) were caught individually, using an immobilization gun (stags) or a drop-net-catch system (hinds) at Glücksburger Heide. The animals were fitted with GSM-GPS collars (Vectronic Aerospace, Pro Light, or GPS PLUS), set to record their position every two hours, and then released.

### Study Design

We used a two-step statistical approach to investigate the spatial behavior of red deer at Glücksburger Heide before, during, and after conservation grazing. We first fitted mixed-effect logistic regression to model red deer use of the conservation grazing sites and to gain insights into how conservation grazing directly affects the likelihood of presence on dry heathland as foraging and mating grounds. In a second modelling approach, we built resource selection functions (RSFs, Manly et al. 2002), using a use-availability design based on mixed-effects logistic regression, in order to explore the effects of conservation grazing on red deer habitat selection on a broader spatial scale. To achieve this aim, we compiled a database consisting of red deer telemetry relocations (used points) and random available points depicting environmental variability (available points) (Boyce and McDonald, 1999; Johnson et al. 2006).

### Red Deer use of Conservation Grazing Sites

Information on how much time red deer need to habituate to disturbances or recover from displacement is often imprecise or varies between different studies. Schultz and Bailey (1978) as well as Edge and Marcum (1985), for instance, describe that habituation can appear “rather rapidly”, depending upon the duration and extent of the disturbance, as well as the history of previous disturbances. Sweeney et al. (1971) report that white-tailed deer (*Odocoileus virginianus*) recover from displacement by hunting dogs as soon as one day after the event, while Sunde et al. (2009) observed that red deer only return to their home ranges six days after a driven hunt. Stewart et al. (2002) state that red deer avoid areas where cattle have grazed for up to seven days. To test for displacement effects, habituation and recovery, we assigned the data to 21-day treatment categories. For statistical analysis, we considered data recorded in the last 21 days before (“before grazing”), the first 21 days (“early grazing”) as well as the next 21 days during (“ongoing grazing”) and the first 21 days after (“after grazing”) each conservation grazing term. We chose the duration of 21 days to consider effects that occur at a temporal scale that is relevant in terms of conservation grazing and wildlife management; and to increase robustness to any potential unknown short-term disturbances, which are not related to conservation grazing (e.g., unofficial recreational activities, small-scale hunting activities). Furthermore, we chose an even number of weeks to account for a potential variation of different (accounted and unaccounted) effects along the course of the week (e.g., recreational activities, traffic) as red deer have been observed to adapt to weekly disturbance regimes (Edge and Marcum 1985, Sibbald et al. 2011). In order to assign the treatment categories, we considered conservation grazing activities at the northern site and the two southern sites separately. Consequently, the assigned treatment category refers to conservation grazing activities at the site(s) which lied within the respective red deer’s home range. Preliminary data exploration showed that the tracked red deer regularly used either the northern grazing site or the two southern grazing sites before conservation grazing started; no individual regularly used both sites. Complying with the study by Sunde et al. (2009), we excluded all data recorded during driven hunts or the following six days. This resulted in a total of 20,160 GPS relocations for 12 red deer (nine hinds, three stags) recorded in 2016, 2017, and 2018 (Tables SI.2, SI.3, SI.4, and Fig. SI.1, supplementary information). To ensure accuracy, all GPS relocations used for later analysis were recorded using at least four satellites (GPS-3D validated, Vectronic Aerospace, Berlin).

The selected data used herein were recorded in summer (May to October) and also in winter (November to January). Red deer display seasonal variations in their spatio-temporal behaviour (Meißner et al. 2012; Ensing et al. 2014), and so in order to account for potentially different responses of red deer to sheep grazing data were split into summer and winter to develop separate seasonal models. The first conservation grazing term in 2016 was only 25 days long; as a result, the winter data only feature three treatment levels (before grazing, early grazing, and after grazing). We fitted two separate logistic regression models for summer and winter. Variables representing different times of the day (day, night, twilight), as well as the rutting and calving periods, were added based on the timestamp of each GPS relocation. A further description and overview of the variables used in the analysis can be found in Table 1.

**Table 1:** Overview of variables used for the different statistical models with a short description of how they were derived

Variable	Description	Type	Model	
			Use	RSF
**Environmental**				
Tree cover	Information on the percentage of tree cover gained from Sentinel-2 satellite imagery with a resolution of 20 meters (Copernicus Land Monitoring Service, 2018).	continuous		**x**
NDVI	(Normalized Difference Vegetation Index, Tucker 1979) Calculations were based on Landsat 8 imagery (band 4 and band 5) recorded in spring. NDVI values were averaged for the years 2015, 2017 and 2018.	continuous		**x**
Distance to conservation-grazing sites	The minimum distance of each used or available location to the closest conservation grazing site in meters.	continuous		**x**
**Temporal**				
Time of the day	Categorized into day, night and twilight based on sunrise, sunset, nautical dusk and nautical dawn, which were computed with the suncalc package (Agafonkin and Thieurmel 2018).	categorical	**x**	**x**
Treatment	Treatment categories “before grazing,” “early grazing,” “ongoing grazing” (only for the summer model) and “after grazing” (each period consisting of 21 days).	categorical	**x**	**x**
Calving	Binary variable representing whether data were recorded during the calving period (May 15^th^ to June 15^th^ according to Bonenfant et al. 2004).	categorical	**x**	**x**
Rutting period	Binary variable representing whether data were recorded during the rutting period (September 1st to October 15^th^ according to Moyes et al. 2011).	categorical	**x**	**x**

Variables were included during model selection for the respective models. The final models did not necessarily include all marked variables (see Tables 2–5)

To investigate how red deer directly use the grazing sites, we employed a mixed-effects logistic regression, using the lme4 package (Bates et al. 2018) in R (version 3.4.4, R Core Team 2018). Use (response) is represented by a binary variable of either 1 or 0, depending on whether a GPS relocation was recorded inside or outside of the conservation grazing areas. The categorical variables time of day (day, night, twilight) and conservation grazing treatment (before grazing, early grazing, ongoing grazing, after grazing), alongside binary variables for the rutting and calving periods (females only), were used as predictors. A random effect representing each individual animal was added to account for individual animal characteristics, social effects (Hebblewhite and Merrill 2008), and unequal amounts of data (Gillies et al. 2006).

Before fitting, we checked all variables for collinearity or multicollinearity by using a Pearson’s correlation and the generalised variance inflation factor (GVIF), respectively. For the winter regression model, the rutting period and calving period variables did not apply. For the summer regression model, we fitted multiple model candidates with and without the rutting or calving period as included effects. The final model was selected by comparing AIC values (Akaike’s Information Criterion, Akaike 1998) and choosing the model with the lowest AIC. We adopted an even more conservative approach than the one suggested by Burnham and Anderson (2004) and deemed a model to be the best one unequivocally if a Δ AIC ≥ 10 was recorded between the top model and the second one (Table SI.5, supplementary information). R² values were calculated using the MuMIn package (Barton 2018), and residual variance was checked using the DHARMa package (Hartig 2018) (Figs. SI.4 and SI.5, supplementary information).

### Resource Selection Functions

We based the seasonal resource selection functions on the same 20,160 GPS relocations selected for the use of conservation grazing areas’ model explained above. Used locations were assigned to treatment categories (before grazing, early grazing, ongoing grazing, after grazing) in the same manner and also featured variables for time of day (day, night, twilight), rutting period, and calving period. The data were then also split into summer and winter. We then expanded this database of GPS relocations by adding available locations. Resource selection functions use recorded locations of animals and locations available to them to investigate whether sites with certain properties are being selected (Boyce and McDonald 1999; Manly et al. 2002; Johnson et al. 2006). In this study, available locations were randomly sampled from each animal’s home range. We computed red deer home ranges based on all available GPS relocations (from 2014 to 2018) for each individual animal, using 0.99 fixed kernel density estimation (KDE). We chose the broader 0.99 KDE instead of 0.95 KDE, which is commonly used for home range modelling, as it was not our goal to consider areas regularly used by the deer but instead to define areas potentially used previously—and therefore available. To determine the optimal ratio of used-to-available locations, following the approach taken by Roberts et al. (2017), we performed a preliminary sensitivity analysis with a subset of the data, which, in our case, yielded an optimal ratio of 1:16 (use: available) locations (Fig. SI.2 and SI.3, supplementary information). Consequently, for each used location, we sampled 16 random locations from the individual deer’s home range, which received the same timestamp as the respective used location.

We then measured distance to the conservation grazing sites for each used and available location. Environmental data representing cover (tree cover) and food resources (Normalised Difference Vegetation Index, NDVI) were assigned to all used and available locations. Previous research has shown that spatial behaviour in large herbivores is mainly governed by a trade-off between the basic needs for forage and cover (Mysterud and Østbye 1999; Godvik et al., 2009). The Normalized Difference Vegetation Index (NDVI; Tucker 1979) describes the vegetation greenness and is regularly used to model the spatial distribution of large herbivores (Pettorelli et al. 2011)—such as red deer (e.g., Hebblewhite et al. 2008; Yankuo et al. 2008; Ranglack et al. 2016). NDVI was found to correlate with above-ground biomass (Borowik et al. 2013) and reflects forage quality (Hebblewhite et al. 2008). Calculations were based on spring satellite imagery to reflect herb and shrub cover prior to full canopy development (Smallidge et al. 2010; Borowik et al. 2013). Oeser et al. (2020) conclude that continuous variables, as used herein, are often more suited than categorical variables in representing resources for large mammals. A further description and overview of the variables used in the analysis can be found in Table 1.

We developed the seasonal RSFs by fitting mixed-effects logistic regressions where use/availability, represented as a binary variable (1/0), was used as a response. Continuous variables representing cover (tree cover), food resources (NDVI) and distance to the conservation grazing sites, as well as the categorical variables time of day (day, night, twilight), treatment (before grazing, early grazing, ongoing grazing, after grazing) and the binary variables for the rutting and calving periods (females only), were used as predictors (Table 1). All continuous variables were scaled in order to fit the models. Individual animal ID was added as a random effect (see section above).

We checked for collinearity or multicollinearity using the Pearson’s correlation and the generalised variance inflation factor (GVIF), respectively. We performed model selection based on AIC (Akaike’s Information Criterion, Akaike 1998) to decide on what variables should be excluded and to detect non-linear relationships (Burnham and Anderson 2004; Bolker et al. 2009; Nakagawa and Schielzeth 2013) (Tables SI.6 and SI.7, supplementary information). The two final models (for winter and summer) were selected by choosing the model with the lowest AIC, if Δ AIC ≥ 10 compared to the model with the next lowest AIC (see previous section). R² values were calculated using the MuMIn package (Barton 2018) which computes R² values for generalized linear mixed models according to Nakagawa et al. (2017), while residual variance was checked by using the DHARMa package (Hartig 2018) (Figs. SI.6 and SI.7, supplementary information).

Finally, significant coefficients (*p* < *0.05*) of the final regression models were used to formulate seasonal resource selection functions, which commonly take an exponential form (Boyce and Waller 2003; Johnson et al. 2006, 2004).

1 $$w\left( x \right) = {{{\mathrm{exp}}}}\left( {\beta _1 \ast x_1 + \beta _2 \ast x_2 + \ldots + \beta _n \ast x_n} \right)$$

Where w(x) is the RSF score, x is a predicting variable and *β* is the respective estimate taken from the logistic regression.

Resource selection functions produce so-called “RSF-scores”, which represent a relative measure of attractiveness to the animals. We validated the seasonal resource selection functions via five-fold cross-validation (Boyce et al. 2002; Koper and Manseau 2012) and by blocked cross-validation, as proposed by Roberts et al. (2017), where folds were blocked by animal. The developed RSFs were used to produce maps of the study area showing the predicted habitat selection of red deer for different scenarios (Johnson et al. 2004; Sawyer et al., 2006; Clarke 2017). This was done using raster files of the environmental variables and the estimated coefficients in the QGIS raster calculator (QGIS Development Team 2018).

## Results

### Red Deer use of the Conservation Grazing Sites

**Fig. 2:**
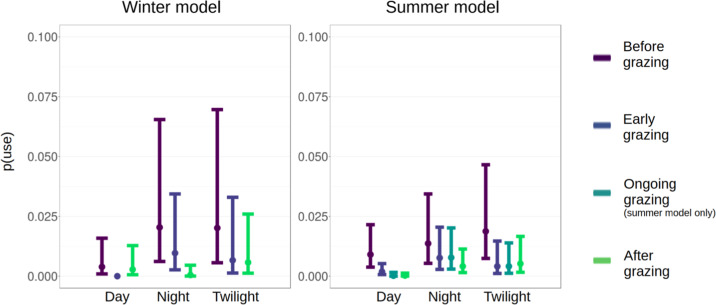
Effects of the different treatments during winter (left) and summer (right) on the direct use of the grazing sites as estimated by logistic regression. The y-axis represents the probability of use by red deer

In summer (Table 2), there was initially a significantly higher probability of use at twilight, but not during the night. During the rutting period, the probability red deer visiting conservation grazing sites significantly increased. All treatments, i.e., “early grazing”, “ongoing grazing” and “after grazing” had a negative effect on the probability of the use of conservation grazing sites by red deer compared to the reference treatment “before grazing”. This led, without exception, to lower probability of use throughout all times of the day. However, this effect seemed to be less pronounced at night, making the use of the sites slightly more probable at night compared to twilight during the treatments “early grazing” and “ongoing grazing”. The probability of use stayed low or decreased further after conservation grazing (Fig. 2, right subplot). The conditional R² (including the random effect) and marginal R² (excluding the random effect) of the model were 0.554 and 0.322, respectively. In the winter model (Table 3), there was initially a significantly higher probability of red deer visiting conservation grazing sites during the night and at twilight. The effects of the treatments “early grazing” and “after grazing” were generally negative. This led to a decreased probability throughout all times of the day for “early grazing”. For “after grazing” this probability recovered during daytime, stayed low at twilight and further decreased at night (Fig. 2, left subplot). The conditional and marginal R² of the model were 0.889 and 0.801, respectively.

**Table 2:** Model summary with estimated coefficients of the model describing the red deer use during summer, effect sizes reported on the link-scale, reference levels: “day,” “before grazing,” “outside rutting period”

	estimate	se	*p*	
(Intercept)	−4.6966	0.4481	<0.0001	***
time of the day–night	0.4165	0.2716	0.1252	
time of the day–twilight	0.7385	0.2695	0.0061	**
treatment – early grazing	−1.5738	0.3377	<0.0001	***
treatment – ongoing grazing	−3.8754	1.0080	0.0001	***
treatment – after grazing	−3.6278	0.7502	<0.0001	***
rut	1.1591	0.2339	<0.0001	***
time of the day – night : treatment – early grazing	0.9866	0.4831	0.0411	*
time of the day – twilight : treatment – early grazing	0.0379	0.6433	0.9530	
time of the day – night : treatment – ongoing grazing	3.3033	1.0578	0.0018	**
time of the day – twilight : treatment – ongoing grazing	2.3462	1.1271	0.0374	*
time of the day – night : treatment – after grazing	2.4229	0.7815	0.0019	**
time of the day – twilight : treatment – after grazing	2.3370	0.8379	0.0053	**
		Random effects		11 (ID)
		Observations		15 001
		Degrees of freedom		14 986
		R² (marginal / conditional)		0.322/0.554

Signif. codes: <0.001***, <0.01**, <0.05*

**Table 3:** Model summary with estimated coefficients of the model describing the red deer use during winter, effect sizes reported on the link-scale, reference levels: “day,” “before grazing,” “outside rutting period”

	estimate	se	*p*	
(Intercept)	−5.5478	0.7242	<0.0001	***
time of the day-night	1.6750	0.4318	0.0001	***
time of the day–twilight	1.6624	0.4874	0.0006	***
treatment—early grazing	−16.6596	20.1575	0.4085	
treatment—after grazing	−0.3281	0.6392	0.6078	
time of the day–night : treatment—early grazing	15.9001	20.1579	0.4302	
time of the day–twilight : treatment–early grazing	15.5364	20.1628	0.4410	
time of the day–night : treatment–after grazing	−3.4450	1.1844	0.0036	**
time of the day–twilight : treatment – after grazing	−0.9410	0.8544	0.2707	
		Random effects		9 (ID)
		Observations		5 159
		Degrees of freedom		5 149
		R² (marginal / conditional)		0.801/0.889

Signif. codes: <0.001***, <0.01**, <0.05*

### Resource Selection Functions

The two seasonal resource selection functions were derived from the AIC-selected logistic regression models according to Eq. 1 (Equations SI.1, SI.2, SI.3, and SI.4, supplementary information). Estimated coefficients of the two logistic regression models can be found in Table 4 (summer model) and Table 5 (winter model).

**Table 4:** Model summary of the logistic regression with estimated coefficients for the summer RSF

	estimate	se	*p*	
(Intercept)	−2.547307	0.028418	<0.0001	***
cover	0.063830	0.024718	0.0010	**
cover²	−0.105948	0.014025	<0.0001	***
NDVI	0.251224	0.012148	<0.0001	***
NDVI²	−0.154700	0.008060	<0.0001	***
Distance to grazing site	−0.383457	0.024396	<0.0001	***
Distance to grazing site²	−0.114533	0.008966	<0.0001	***
treatment—early grazing	−0.022268	0.032467	0.4928	
treatment—ongoing grazing	0.003820	0.033589	0.9095	
treatment—after grazing	−0.079469	0.037543	0.0343	*
time of the day–night	0.092512	0.045967	0.0442	*
time of the day–twilight	0.108984	0.047094	0.0207	*
calving	−0.134478	0.028494	<0.0001	***
*cover* : *treatment*—*early grazing*	0.297816	0.033271	<0.0001	***
*cover* : *treatment*—*ongoing grazing*	0.306423	0.034406	<0.0001	***
*cover* : *treatment*—*after grazing*	0.489362	0.038622	<0.0001	***
*cover* : *time of the day-night*	−0.562642	0.045959	<0.0001	***
*cover* : *time of the day*–*twilight*	−0.290562	0.048337	<0.0001	***
*treatment–early grazing* : *time of the day-night*	−0.091212	0.067028	0.1736	
*treatment - ongoing grazing* : *time of the day-night*	−0.188606	0.064811	0.0036	**
*treatment - after grazing* : *time of the day-night*	0.039193	0.061801	0.5260	
*treatment–early grazing* : *time of the day–twilight*	0.018498	0.069750	0.7909	
*treatment - ongoing grazing* : *time of the day–twilight*	−0.053928	0.072101	0.4545	
*treatment - after grazing* : *time of the day–twilight*	−0.124201	0.086467	0.1509	
*distance to grazing site* : *treatment–early grazing*	0.323440	0.033758	<0.0001	***
*distance to grazing site* : *treatment–ongoing grazing*	0.592911	0.034296	<0.0001	***
*distance to grazing site* : *treatment–after grazing*	0.530440	0.036754	<0.0001	***
*distance to grazing site* : *time of the day-night*	0.502155	0.048055	<0.0001	***
*distance to grazing site* : *time of the day–twilight*	0.353136	0.051951	<0.0001	***
*cover* : *calving*	0.424647	0.029797	<0.0001	***
*cover* : *treatment–early grazing* : *time of the day-night*	−0.478894	0.066460	<0.0001	***
*cover* : *treatment - ongoing grazing* : *time of the day-night*	−0.465471	0.063703	<0.0001	***
*cover* : *treatment - after grazing* : *time of the day-night*	−0.341177	0.062112	<0.0001	***
*cover* : *treatment–early grazing* : *time of the day–twilight*	−0.261897	0.070833	0.0002	***
*cover* : *treatment - ongoing grazing* : *time of the day–tiwilight*	−0.279425	0.071666	<0.0001	***
*cover* : *treatment - after grazing* : *time of the day–twilight*	0.025574	0.081728	0.7543	
*distance to grazing site* : *treatment–early grazing* : *time of the day-night*	−0.240115	0.067064	0.0003	***
*distance to grazing site* : *treatment - ongoing grazing* : *time of the day-night*	−0.341413	0.064289	<0.0001	***
*distance to grazing site* : *treatment - after grazing* : *time of the day-night*	−0.390236	0.062908	<0.0001	***
*distance to grazing site* : *treatment–early grazing* : *time of the day–twilight*	−0.062028	0.075086	0.4088	
*distance to grazing site* : *treatment - ongoing grazing* : *time of the day–twilight*	−0.195044	0.075898	0.0102	*
*distance to grazing site* : *treatment - after grazing* : *time of the day–twilight*	0.140573	0.085334	0.0995	
		Random effects		11 (ID)
		Observations		255 017
		Degrees of freedom		254 974
		R² (marginal / conditional)		0.118/0.118

Signif. codes: <0.001***, <0.01**, <0.05*

**Table 5:** Model summary of the logistic regression with estimated coefficients for the winter RSF

	estimate	se	*p*	
(Intercept)	- 3.1470126	0.0923909	<0.0001	***
cover	0.3096964	0.0540430	<0.0001	***
cover²	- 0.3505960	0.0239475	<0.0001	***
NDVI	0.2267749	0.0160572	<0.0001	***
Distance to grazing site	−1.7420097	0.0752500	<0.0001	***
Distance to grazing site²	−0.3897231	0.0234184	<0.0001	***
treatment−early grazing	0.5336198	0.1030164	<0.0001	***
treatment—after grazing	0.3694910	0.0995824	0.0002	***
time of the day-night	0.7121565	0.0862280	<0.0001	***
time of the day—twilight	0.2590906	0.1207299	0.0319	*
cover : treatment–early grazing	0.0622428	0.0832894	0.4549	
cover : treatment - after grazing	0.0829775	0.0767361	0.2795	
cover : time of the day–night	−1.0059007	0.0685708	<0.0001	***
cover : time of the day–twilight	−0.2882686	0.0909075	0.0015	**
treatment–early grazing : time of the day–night	−0.3335367	0.1191349	0.0051	**
treatment - after grazing : time of the day–night	−0.2015672	0.1142131	0.0776	
treatment–early grazing : time of the day–twilight	−0.0951990	0.1661539	0.5667	
treatment - after grazing : time of the day–twilight	−0.0297798	0.1586907	0.8511	
distance to grazing site : treatment–early grazing	0.6890899	0.1029629	<0.0001	***
distance to grazing site : treatment–after grazing	0.4668890	0.0964969	<0.0001	***
distance to grazing site : time of the day–night	1.3263162	0.0847477	<0.0001	***
distance to grazing site : time of the day–twilight	0.2427902	0.1168348	0.0377	*
cover : treatment–early grazing : time of the day–night	0.2903048	0.1032598	0.0049	**
cover : treatment - after grazing : time of the day–night	0.5652130	0.0949744	<0.0001	***
cover : treatment–early grazing : time of the day–twilight	0.0855984	0.1399958	0.5409	
cover : treatment - after grazing : time of the day–twilight	−0.0001537	0.1279047	0.9990	
distance to grazing site : treatment–early grazing : time of the day–night	−0.4220144	0.1234568	0.0006	***
distance to grazing site : treatment - after grazing : time of the day–night	−0.5319586	0.1157901	<0.0001	***
distance to grazing site : treatment–early grazing : time of the day–twilight	−0.0549513	0.1729694	0.7507	
distance to grazing site : treatment - after grazing : time of the day–twilight	0.0172922	0.1612649	0.9146	
		Random effects		9 (ID)
		Observations		87 703
		Degrees of freedom		87 672
		R² (marginal / conditional)		0.389/0.393

Signif. codes: <0.001***, <0.01**, <0.05*

Both seasonal RSFs performed satisfactorily during random cross-validation (Spearman’s rho: 0.973 and 0.985). During the stricter blocked cross-validation, the summer RSF performed worse (Spearman’s rho: 0.891)—as expected compared to random cross-validation (Roberts et al. 2017)—while the performance of the winter RSF actually improved compared to the random cross-validation (Spearman’s rho: 0.992). Results from the two cross-validation approaches are visualized in Figs. SI.8, SI.9, SI.10, and SI.11 (supplementary information).

**Fig. 3:**
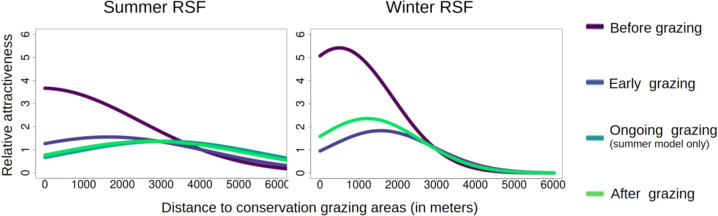
Predicted selection for distance to grazing sites during summer (left) and winter (right) during the day. The x-axis represents distance to the conservation grazing site (meters), and relative attractiveness is shown on the *y*-axis. For visualization purposes, scores produced by the RSF were scaled by dividing them by the scenarios-specific median

**Fig. 4:**
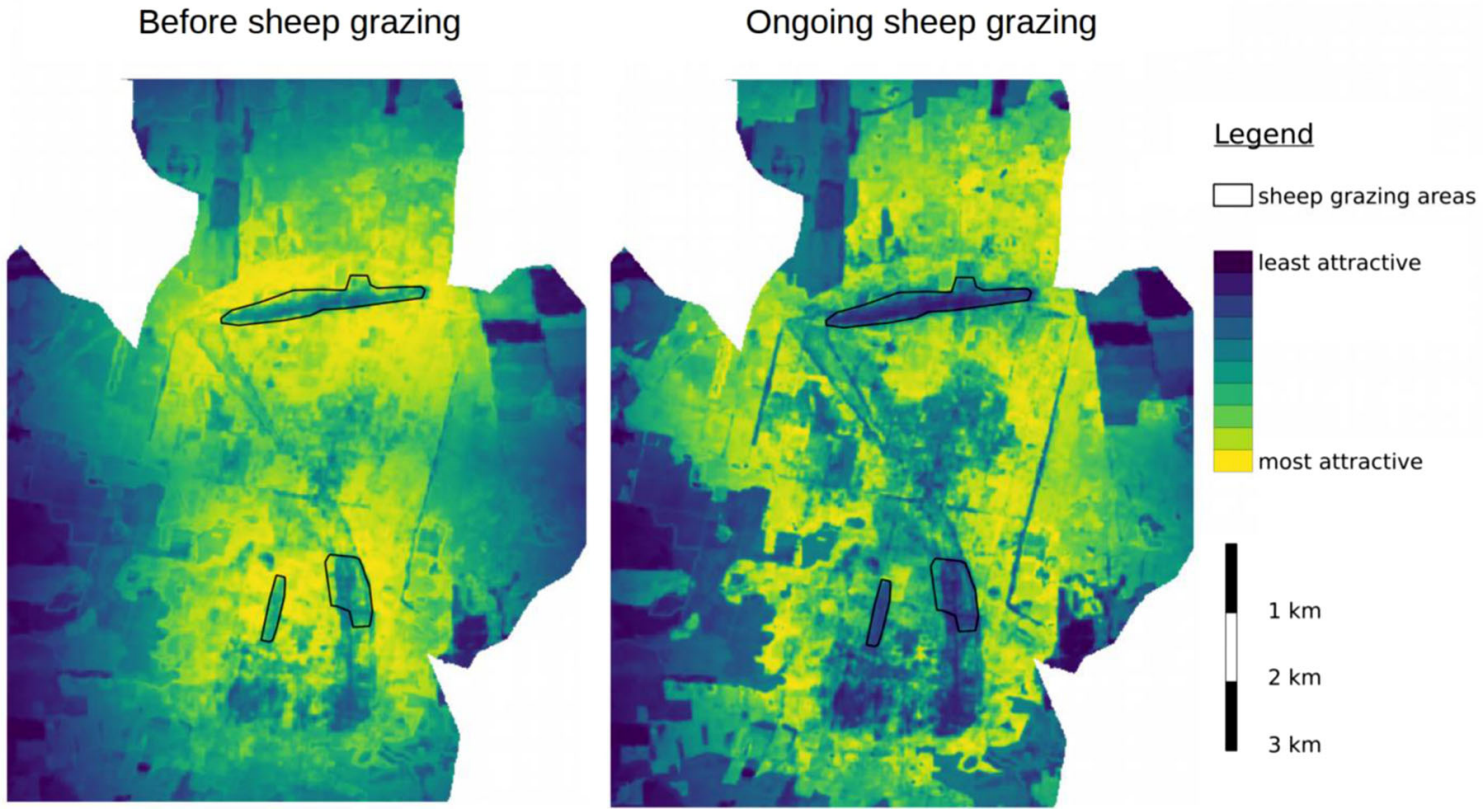
Maps of the study area depicting the spatial distribution of RSF scores before conservation grazing (left) and during the later stages of conservation grazing (right) during the summer and in the daytime

RSF scores for the summer RSF, when calculated and plotted along a gradient of “distance to conservation grazing site,” indicated that for all times of the day (day, night, twilight), areas close to the conservation grazing sites were preferred the most before sheep grazed there (Fig. SI.12, supplementary information). As sheep-grazing started, red deer presence shifted further away and continued to do so in line with ongoing conservation grazing; after conservation grazing, it either remained static or slightly shifted back within the 3 weeks considered here. The most striking change in preference occurred during the daytime (Fig. 3), while the effect was smallest during the night. Plotted RSF scores for winter reveal a similar trend, i.e., the highest preference for areas near the conservation grazing sites being before sheep-grazing started (Fig. SI.13, supplementary information). With the advent of sheep-grazing, the deer moved further away from the conservation grazing sites, and after sheep-grazing ceased, their presence partly (day, twilight) or fully (night) recovered. As in the summer RSF, this effect was most pronounced during the day and at its weakest at night. A comparison of the summer and winter RSFs shows that the initial selection of areas close to conservation grazing sites was stronger in winter. When comparing habitat selection before conservation grazing and during the other treatments, changes are visible up to 3000 m away from the grazing sites in winter, and more than 3000 m in summer. Spatially visualized RSF scores (RSF maps) are presented in Fig. 4.

## Discussion

Our modelling approach highlights the significant effects of conservation grazing on the spatio-temporal behavior of red deer, leading to their temporal displacement from conservation grazing sites and adjacent areas up to a distance of 3000 m. Following the start of conservation grazing, the use of these sites by red deer decreases not only during the day, but also during the night and at twilight when sheep are temporarily absent; it also remains low during the first three weeks after sheep-grazing ceases. Our seasonal resource selection functions indicate that red deer regularly use conservation grazing sites and their surroundings at Glücksburger Heide during the day and at twilight in both summer and winter, but this use is reduced considerably during conservation grazing and the three weeks following these grazing activities.

This suggests that sheep-grazing with herded sheep is perceived by red deer as a direct disturbance. A wandering flock of sheep, including a shepherd and sheepdogs, represents a complex combination of multiple visual, acoustic, and olfactory stimuli. Sheepdogs especially move quickly and change direction often when working with sheep, and so their movements are difficult to predict and they are most likely perceived as a predatory threat by red deer. But also walking humans have been reported to represent a significant source of disturbance (Stankowich 2008). Moreover, the open areas used for sheep-grazing provide little cover for red deer, and it is known that they are specifically sensitive to disturbances in open areas (Stankowich 2008; Jayakody et al. 2011). Consequently, they may avoid these areas during conservation grazing, due to a combination of this disturbance and a lack cover in which to hide. Our finding that the effects of conservation grazing reach beyond the limits of the actual grazing sites into more covered areas suggests that acoustic and probably olfactory stimuli also affect red deer regardless of the available cover. An alternative explanation for these far-ranging effects could be that red deer select other open areas as a consequence of displacement, thereby resulting in a general spatial shift of their home ranges (Peek et al. 1982; van Dyke and Klein 1996). Edge and Marcum (1985) report similar far-reaching displacement effects of logging operations on red deer, in that they remained a mean distance of 2000m away from logging operations and did not move closer than 500 to 1000 m. One additional indirect effect of conservation grazing on red deer could be related to wolves present in the study area. These might be attracted by the livestock leading to an increased presence of wolves in the vicinity of conservation grazing activities, which in turn increases general vigilance of red deer and triggers avoidance of these areas (van Beeck Calkoen et al. 2021).

The effects observed by us appear to be more pronounced in winter than in summer. The initial use of the sites indicated by logistic regression, as well as the selection of these sites and their surroundings implied by the RSF, were greater during winter. We assume that the large open heathland areas at Glücksburger Heide play a more critical role as grazing sites for red deer in winter, as there are fewer alternative agricultural food sources around the study area at that time of the year. The increased hunting pressure, especially in the surrounding areas, could also be a contributing factor. The importance of heathland areas as foraging grounds for red deer in winter is also underlined by the findings of Riesch et al. (2019), who observed significantly greater forage removal rates in heathland at this time of year. Thus, the overall impact of sheep-grazing on red deer in Glücksburger Heide can be assumed to be greater in winter. Nevertheless, we must be careful when interpreting the statistical output of our winter logistic regression. The patterns seem less clear with generally larger p-values (Table 3) as significances in this model might be restrained by the smaller sample size of the winter models (Demidenko 2007).

Although other studies mention red deer habituating to disturbances (Thompson and Henderson 1998; Found and St. Clair 2016), we found no indication for such habituation during periods of conservation grazing with sheep and associated disturbance stimuli. However, it should be noted that we only tested for a short-term habituation effect during conservation grazing in winter. During summer, where we also analyzed the spatio-temporal behavior of red deer during later stages of conservation grazing, displacement effects increased or remained stable rather than decreased – as one would assume in the case of habituation. Both the direct use of the sites and the selection of their surroundings (RSFs) decreased at all times of the day (day, night, twilight) when conservation grazing started and continued (summer). These effects seems to be relatively less pronounced during the night suggesting direct disturbance effects of conservation grazing during daytime; but this could also indicate a general shift to increased nocturnal activities due to generally higher disturbance levels (Ensing et al. 2014). We found no indication of any short-term spatio-temporal adaption to disturbance as described by Edge and Marcum (1985) in the case of logging activities, or by Westekemper et al. (2018) and Marion et al. (2021) in the case of human recreational activities. There were also no signs of any compensatory use of the conservation grazing sites by red deer at night or at twilight, when sheep were not present. Our findings are in line with those of Sibbald et al. (2011), who did not observe any compensatory use of areas around hiking tracks during the nightly absence of hill-walkers. There are several possible explanations for this observation in the case of conservation grazing at Glücksburger Heide. We suspect that, in addition to any direct disturbance effects, sheep-grazing temporarily reduces the attractiveness of affected areas for red deer. Stewart et al. (2002) report correspondingly that red deer avoid areas for at least seven days where cattle have grazed. Forage depletion caused by feeding and trampling sheep might be one reason for reduced attractiveness in this case (Bakker et al. 1983; Jauregui et al. 2009), but another cause might be lingering olfactory stimuli, since Chabot et al. (1996) found that the scent of sheep and humans can reduce the general palatability of vegetation for red deer, while canid (*Canidae*) scent can even provoke symptoms of physical stress or lead to avoidance of the affected areas (van Beeck Calkoen et al. 2021). This might be a crucial factor in why red deer also reduce their use of these sites during the night and at twilight when sheep are not present. An additional explanation might be spatial memory, which is an emerging field in behavioral ecology (Fagan et al. 2013). In this regard, there is evidence that spatial memory plays an important role for the use of resources by red deer (Gautestad et al. 2013), and it is likely that it also plays an important role in the avoidance of disturbances. It might even outweigh a red deer’s ability to assess the temporal dynamics of such, especially if they are temporally difficult to predict. As a consequence, they will avoid the affected areas for a certain time, even if the reason for avoiding it in the first place is no longer present.

Within the considered time period (21 days), red deer do not resort to their original spatio-temporal behavior after conservation grazing stops. Their use of the conservation grazing sites remains low at all times of the day in summer but partially recovers during the daytime in winter. We make a similar observation for habitat selection in terms of distance to conservation grazing sites: Selection is low after conservation grazing ceases during the summer but partly recovers in the winter. One important factor could be the overall greater selection of open areas by red deer, leading to faster re-utilization in winter. However, the observed seasonal differences might also be induced by additional factors such as the removal of tree saplings during summer or increased hunting pressure during winter, as well as varying amounts of food resources in the surrounding areas throughout the year. We conclude that the effects of conservation grazing on the spatio-temporal behavior of red deer in Glücksburger Heide persist for some time after conservation grazing activities stop. However, the design of this study does not allow us to make clear inferences about the exact temporal dimensions of enduring behavioral changes. Nonetheless, it should be noted that during the following 21 days, both the direct presence on the sites and the overall habitat selection significantly differ from what is observed before conservation grazing. This delay in revisiting these areas might be caused by the already mentioned depletion of forage vegetation, remaining scents, or persistent spatial memory of the disturbance, the latter of which might push these animals to other suitable areas they continue to use after sheep-grazing has stopped. Aspects of spatial familiarity (Piper 2011) might also play a role, as red deer have been found to prefer areas with which they are familiar and return to well-known foraging grounds on a regular basis (Wolf et al. 2009).

Our findings are supported by good results during the two different cross-validation approaches. The resemblance of the overall patterns between the different seasonal models provides additional confidence that our results reflect the veridical effects of conservation grazing on the spatio-temporal behavior of red deer at Glücksburger Heide. Nevertheless, the structural composition of a landscape and the overall availability of different land-cover types can essentially influence general spatial behavior (“functional responses”): Godvik et al. 2009; Matthiopoulos et al. 2011 and thus also responses to disturbance (Hebblewhite and Merrill 2008). We, therefore, suspect that the impact of conservation grazing on red deer is variable and depends on the surrounding conditions.

Our findings indicate that interactions between wild and domesticated herbivores should be considered in conservation planning for semi-natural open areas such as dry heath- or grasslands. Both conservation grazing with domesticated herbivores (Dostálek and Frantík 2008; Jauregui et al. 2009) and grazing by red deer (Riesch et al. 2019, 2020) have been found to increase plant species diversity, in order to reduce tree encroachment and to maintain the open character of such areas. In this study, however, we demonstrate that conservation grazing with sheep herding– at least temporarily – is capable of displacing wild red deer. As a result, the beneficial effects of wild ungulate herbivores and conservation grazing might compete when applied simultaneously. On the other hand, mixed grazing regimes have on other occasions proven to be especially efficient and to promote biodiversity (Rosa García et al. 2013; Fraser et al. 2014; Marchetto et al., 2021). This could also be the case for a combination of sheep and wild ungulate herbivores such as red deer. In the anthropogenic landscape, where red deer can be attracted by other food sources such as surrounding agricultural areas, grazing with shepherded sheep can be applied in a more targeted manner. In contrast, wild red deer are less restricted by property and management boundaries and can help to counteract encroachment at forest edges and other transitional landscape elements. Other relevant consequences of the displacement of red deer from the conservation grazing sites might be related to aspects of wildlife management. It is very difficult to precisely predict any population-level effects for red deer. Agricultural areas in the surroundings probably provide sufficient though temporally shifting food sources. However, the (temporary) loss of relatively undisturbed open foraging grounds might increase red deers’ perception of risks and flight responses - especially for females or groups with young offspring (Stankowich 2008). Since the displacement effects seem to reach beyond the conservation grazing sites and affect larger areas we also expect spatial shifts in human-wildlife conflicts with forestry (Bobrowski et al. 2020), agriculture (Walter et al. 2010) or traffic (Mysterud 2004) and hunting activities might have to be adapted. All these aspects need to be considered and pondered in order to optimize the outcome. One solution could be the careful timing of grazing with herded livestock to reduce displacement of wild ungulate herbivores. In order to optimize mixed grazing schemes better mechanistic understanding of the effects of conservation grazing on wild herbivores is needed. Therefore future studies should aim to disentangle the effects of the presence of domesticated animals, dogs, and shepherds, as well as of resulting resource depletion.

## Supplementary information

Supplementary Information

## Footnotes

1 The term ‘driven hunt’ refers to larger scale hunting activities: One or several groups of hunters and dogs move through an area to systematically push the targeted game species out of cover. A number of hunters/shooters are positioned along the anticipated directions of escape to target the evading game.
