# Supplementary material for: Displacement Effects of Conservation Grazing on Red Deer (Cervus elaphus) Spatial Behaviour
Source: Environ Manage. 2022 Aug 22;70(5):763–79. doi: 10.1007/s00267-022-01697-6 (PMC9519651; doi:10.1007/s00267-022-01697-6)
Supplement: Supplementary file 1 — Supplementary Information [file 267_2022_1697_MOESM1_ESM.pdf]

Supplementary Information to

Displacement effects of conservation grazing on red deer (*Cervus elaphus*) spatial behaviour

Fabio Weiss, Frank U. Michler, Benjamin Gillich, Jörg E. Tillmann, Simone Ciuti, Marco Heurich and Siegfried Rieger

Environmental Management (Springer Nature)

University for Sustainable Development Eberswalde, fabio.weiss@hnee.de

A) Supplementary tables

Table SI.1 Conservation grazing terms at Glücksburger Heide, grazing sites listed together were used simultaneously; [a] for exact site location refer to Fig. 1 of the main article, [b] cut-off date for data included in the analysis, conservation grazing was still ongoing.

Site[a]	From	To
South-west / south-east	08.12.2016	21.12.2016
North	10.06.2017	23.07.2017
South-west / south-east	23.07.2017	26.09.2017
South-west / south-east	01.06.2018	03.09.2018
North	04.09.2018	16.10.2018[b]

Table SI.2 GPS collared red deer selected for analysis. Site affiliation refers conservation sites that are presented in Fig. 1 of the main article. Animals without end date were still transmitting at the time of data analysis.

Animal ID	Sex	Age	Site affiliation	Start	End
3	f	Adult	South	14.01.2015	
9	f	Adult	South	14.01.2015	
10	f	Adult	South	14.01.2015	
11	f	Adult	South	14.01.2015	
13	f	Adult	South	14.01.2015	
16	f	Subadult (2016)	South	14.01.2015	25.01.2018
40	m	Adult	South	19.09.2016	
20	m	Adult	South	26.09.2016	
5_2	m	Adult	South	26.09.2016	11.02.2017
34	f	Adult	North	05.03.2017	
37	f	Adult	North	05.03.2017	
44_3	f	Subadult (2018)	North	03.03.2017	

Table SI.3 Overview of red deer GPS relocations selected for analysis. Treatment categories and time periods relate to conservation grazing on the northern site.

				GPS relocations		
from	to	*treatment*	season	ID 34	ID 37	ID 44_3
18.05.2017	09.06.2017	*pre-grazing*	summer	258	260	-
10.06.2017	01.07.2017	*early grazing*	summer	249	250	-
02.07.2017	23.07.2017	*ongoing grazing*	summer	252	250	-
24.07.2017	14.08.2017	*post-grazing*	summer	269	248	-
13.08.2018	03.09.2018	*pre-grazing*	summer	140	257	54
04.09.2018	25.09.2018	*early grazing*	summer	151	140	-
26.09.2018	17.10.2018	*ongoing grazing*	summer	42	133	-
-	-	*post-grazing*	summer	-	-	-

Table SI.4 Overview of red deer GPS relocations selected for analysis. Treatment categories and time periods relate to conservation grazing on two southern sites.

								GPS relocations				
from	to	*treatment*	season	ID 3	ID 9	ID 10	ID 11	ID 13	ID 16	ID 20	ID 40	ID 5_2
09.11.2016	07.12.2016	*pre-grazing*	winter	245	260	258	179	111	251	256	259	251
08.12.2016	21.12.2016	*early grazing*	winter	161	140	164	77	82	162	162	165	156
-	-	*ongoing grazing*	winter	-	-	-	-	-	-	-	-	-
22.12.2016	11.01.2017	*post-grazing*	winter	236	248	242	183	83	245	246	249	88
01.07.2017	22.07.2017	*pre-grazing*	summer	247	259	243	199	170	249	70	262	-
23.07.2017	14.08.2017	*early grazing*	summer	255	273	256	209	152	264	-	272	-
15.08.2017	05.09.2017	*ongoing grazing*	summer	240	260	248	205	191	252	76	258	-
27.09.2017	17.10.2017	*post-grazing*	summer	227	249	234	175	165	241	81	246	-
10.05.2018	31.05.2018	*pre-grazing*	summer	227	258	243	193	149	-	-	261	-
01.06.2018	22.06.2018	*early grazing*	summer	236	258	239	186	147	-	-	262	-
23.06.2018	14.07.2018	*ongoing grazing*	summer	225	260	237	195	166	-	-	260	-
04.09.2018	24.09.2018	*ongoing grazing*	summer	232	248	240	193	159	-	-	246	-

Table SI.5 AIC-based model selection for the model describing red deer use during summer. Model candidates were considered supported if △ AIC ≥ 10 compared to the model candidate with the next lowest AIC value.

	`glmer` model term	AIC	ΔAIC	R^2_c	R^2_m	supported
$\mathbf{m1}_{summer}$	$Utilisation\ of\ grazing\ sites \sim time\ of\ the\ day + treatment + time\ of\ the\ day * treatment + (1 \mid animal\ ID)$	1665.480	-	0.305	0.550	-
$\mathbf{m2}_{summer}$	$\mathbf{m1}_{summer} + rut$	1643.562	-21.918	0.322	0.554	yes
$\mathbf{m3}_{summer}$	$\mathbf{m2}_{summer} + calving$	1638.565	-4.997	0.323	0.558	no

Table SI.6 AIC-based model selection for the logistic regression used to derive coefficients of the summer RSF. Model candidates were considered supported if △ AIC ≥ 10 compared to the model candidate with the next lowest AIC value.

	`glmer` model term	AIC	ΔAIC	R_c^2	R_m^2	supported
$\mathbf{m1}_{summerRSF}$	$Selection\ of\ location \sim cover + NDVI + distance\ to\ grazing\ site + cover \ldots * treatment * time\ of\ the\ day + distance\ to\ grazing\ site \ldots * treatment * time\ of\ the\ day + (1 \mid animal\ ID)$	111044	-	0.072	0.072	-
$\mathbf{m2}_{summerRSF}$	$\mathbf{m1}_{summerRSF} + cover^2$	110919.9	-124.1	0.080	0.080	yes
$\mathbf{m3}_{summerRSF}$	$\mathbf{m2}_{summerRSF} + distance\ to\ grazing\ site^2$	110711	-208.9	0.090	0.090	yes
$\mathbf{m4}_{summerRSF}$	$\mathbf{m3}_{summerRSF} + NDVI^2$	110284.1	-426.9	0.112	0.112	yes
$\mathbf{m5}_{summerRSF}$	$\mathbf{m4}_{summerRSF} + cover * calving$	110073.2	-210.9	0.118	0.118	yes
$\mathbf{m6}_{summerRSF}$	$\mathbf{m5}_{summerRSF} + cover * rut$	110077.1	+3.9	0.118	0.118	no

Table SI.7 AIC-based model selection for the logistic regression used to derive coefficients of the winter RSF. Model candidates were considered supported if △ AIC ≥ 10 compared to the model candidate with the next lowest AIC value.

	`glmer` model term	AIC	ΔAIC	R_c^2	R_m^2	supported
$\mathbf{m1}_{winterRSF}$	$Selection\ of\ location \sim cover + NDVI + distance\ to\ grazing\ site + cover \ldots * treatment * time\ of\ the\ day + distance\ to\ grazing\ site \ldots * treatment * time\ of\ the\ day + (1 \mid animal\ ID)$	36487.31	-	0.234	0.238	-
$\mathbf{m2}_{winterRSF}$	$\mathbf{m1}_{winterRSF} + cover^2$	36306.36	-180.95	0.248	0.251	yes
$\mathbf{m3}_{winterRSF}$	$\mathbf{m2}_{winterRSF} + distance\ to\ grazing\ site^2$	35968.27	-338.09	0.389	0.393	yes
$\mathbf{m4}_{winterRSF}$	$\mathbf{m3}_{winterRSF} + NDVI^2$	35964.05	-4.22	0.389	0.393	no

B) Supplementary equations

Equations SI.1 and SI.2 Resource Selection Function for the summer; base function for the reference levels (equation SI.2) with the scenario-specific extensions (equation SI.2).

$$
\begin{aligned}
w = exp(\ & 0.063830 * cover \\
& - 0.105948 * cover^2 \\
& + 0.251224 * NDVI \\
& - 0.154700 * NDVI^2 \\
& - 0.383457 * distance\ to\ grazing\ site \\
& - 0.114533 * distance\ to\ grazing\ site^2 \qquad (1)
\end{aligned}
$$

$$
\begin{aligned}
& - 0.079469 * aftergrazing \\
& + 0.092512 * night \\
& + 0.108984 * twilight \\
& - 0.134478 * calving \\
& + 0.297816 * cover * early\ grazing \\
& + 0.306423 * cover * ongoing\ grazing \\
& + 0.489362 * cover * aftergrazing \\
& - 0.562642 * cover * night \\
& - 0.290562 * cover * twilight \\
& - 0.188606 * ongoing\ grazing * night \\
& + 0.323440 * distance\ to\ grazing\ site * early\ grazing \\
& + 0.592911 * distance\ to\ grazing\ site * ongoing\ grazing \\
& + 0.530440 * distance\ to\ grazing\ site * aftergrazing \\
& + 0.502155 * distance\ to\ grazing\ site * night \\
& + 0.353136 * distance\ to\ grazing\ site * twilight \\
& + 0.424647 * cover * calving \\
& - 0.478894 * cover * early\ grazing * night \\
& - 0.465471 * cover * ongoing\ grazing * night \\
& - 0.341177 * cover * aftergrazing * night \\
& - 0.261897 * cover * early\ grazing * twilight \\
& - 0.279425 * cover * ongoing\ grazing * twilight \\
& - 0.240115 * distance\ to\ grazing\ site * early\ grazing * night \\
& - 0.341413 * distance\ to\ grazing\ site * ongoing\ grazing * night \\
& - 0.390236 * distance\ to\ grazing\ site * aftergrazing * night \\
& - 0.195044 * distance\ to\ grazing\ site * ongoing\ grazing * twilight) \qquad (2)
\end{aligned}
$$

Equations SI.3 and SI.4 Resource Selection Function for the winter; base function for the reference levels (equation SI.3) with the scenario-specific extensions (equation SI.4).

$$
\begin{aligned}
w = exp(\; & 0.3096964 * cover \\
& - 0.3505960 * cover^2 \\
& + 0.2267749 * NDVI \\
& - 1.7420097 * distance\ to\ grazing\ site \\
& - 0.3897231 * distance\ to\ grazing\ site^2
\end{aligned}
\tag{3}
$$

$$
\begin{aligned}
& + 0.5336198 * earlygrazing \\
& + 0.3694910 * aftergrazing \\
& + 0.7121565 * night \\
& + 0.2590906 * twilight \\
& - 1.0059007 * cover * night \\
& - 0.2882686 * cover * twilight \\
& - 0.3335367 * grazing * night \\
& + 0.6890899 * distance\ to\ grazing\ site * earlygrazing \\
& + 0.4668890 * distance\ to\ grazing\ site * aftergrazing \\
& + 1.3263162 * distance\ to\ grazing\ site * night \\
& + 0.2427902 * distance\ to\ grazing\ site * twilight \\
& + 0.2903048 * cover * earlygrazing * night \\
& + 0.5652130 * cover * aftergrazing * night \\
& - 0.4220144 * distance\ to\ grazing\ site * earlygrazing * night \\
& - 0.5319586 * distance\ to\ grazing\ site * aftergrazing * night
\end{aligned}
\tag{4}
$$

C) Supplementary Fig.

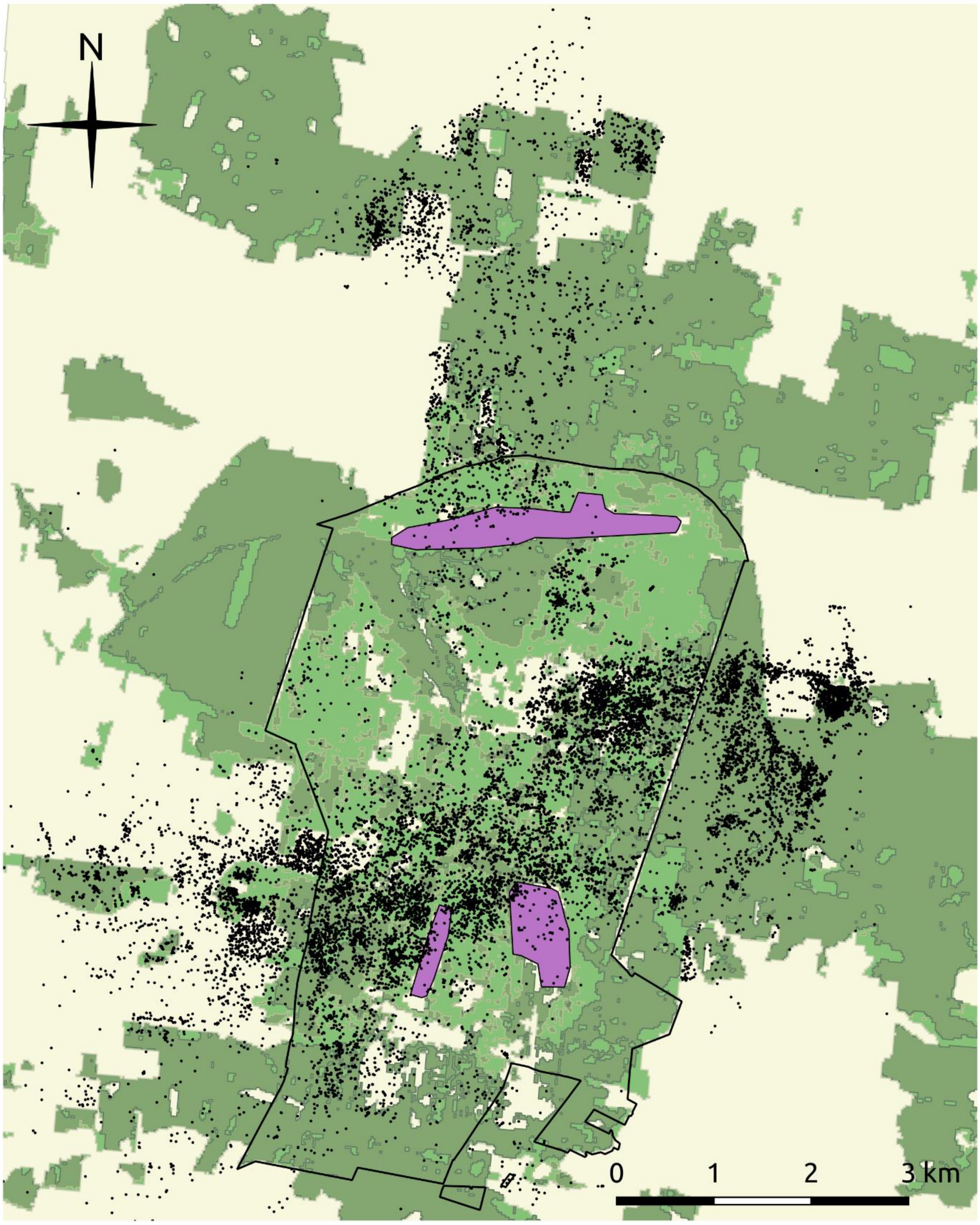

Fig. SI.1 GPS relocations of 12 red deer in Glucksburger Heide analysed in this study. Lightgreen and darkgreen represent deciduous and coniferous forest, respectively (Foresttype product HRL, Copernicus Land Monitoring Service 2018). Black lines mark the limit of the DBU-managed conservation area, grazing sites are highlighted in purple.

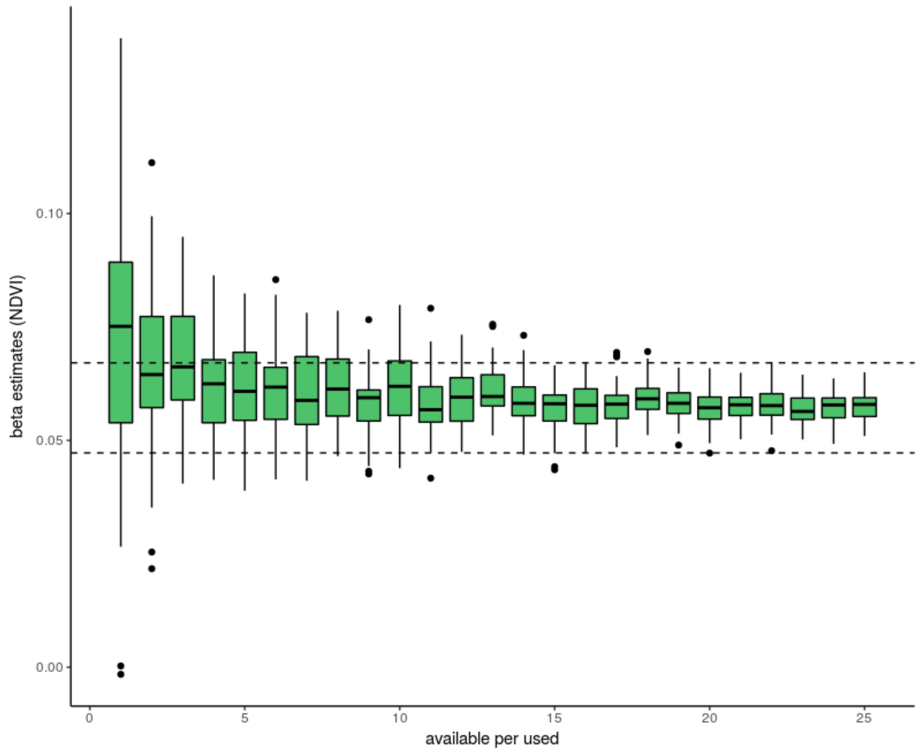

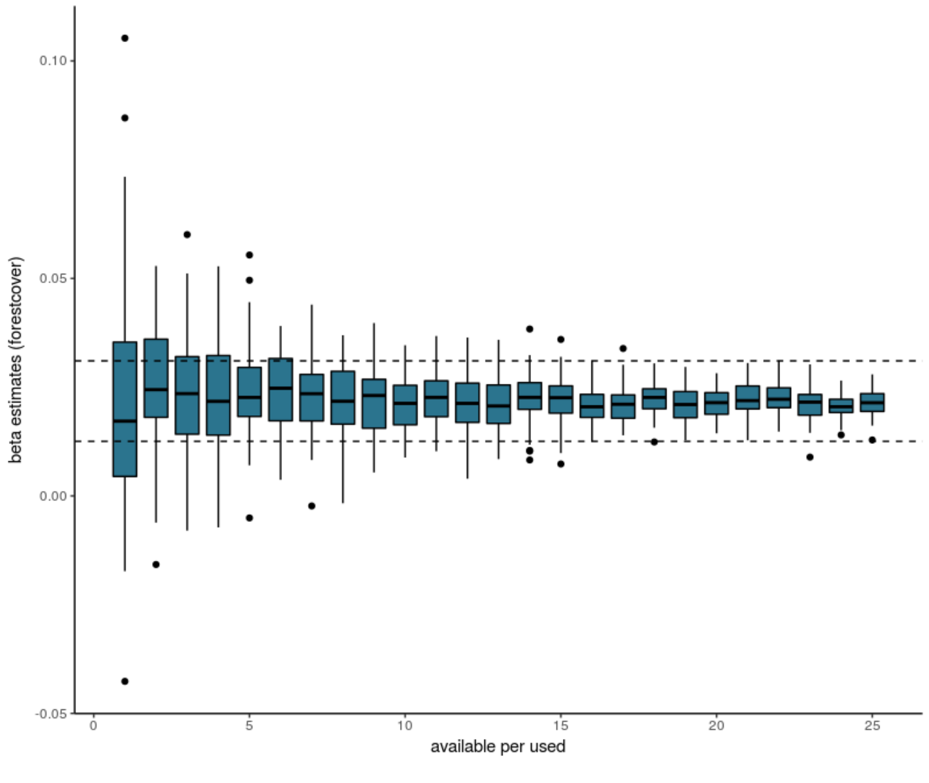

Fig. SI.2 & SI.3 We performed a sensitivity analysis to assess the optimal ratio of used and available locations for the RSF. For that matter, a Generalized Linear Model (GLM) including NDVI and forest-cover as predictors was run for a subset of the data. The subset consisted of 1,783 used locations of one hind (ID 11) and available locations randomly sampled from its availability range. The GLM was run a total of 1,200 times - 50 runs each for 25 different used-available ratios (1:1 to 1:25) with each run of the same ratio sourcing from a new random draw of available locations. Presented Fig. show the results of the sensitivity analysis: **Fig. SI.2 (top)** shows beta-estimates for NDVI**; Fig. SI.3 (bottom)** shows beta-estimates for cover. Dotted lines mark the fluctuation of β estimates at the selected ratio of 1:16.

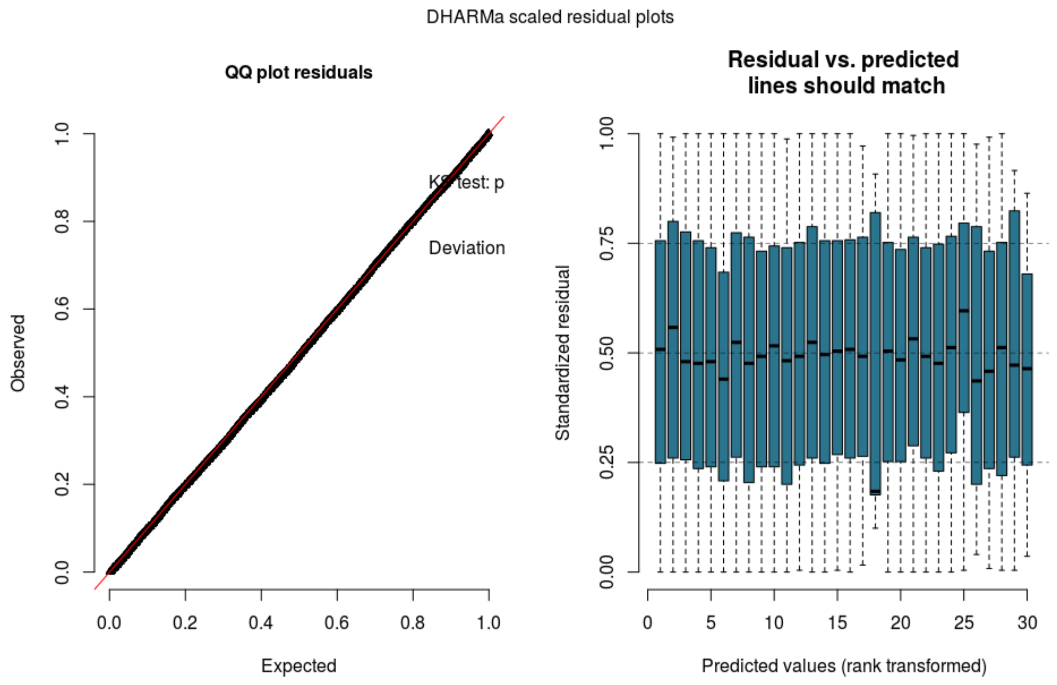

Fig. SI.4 DHARMa diagnostic residual plots of the logistic regression modelling the red deer use of conservation grazing sites in summer.

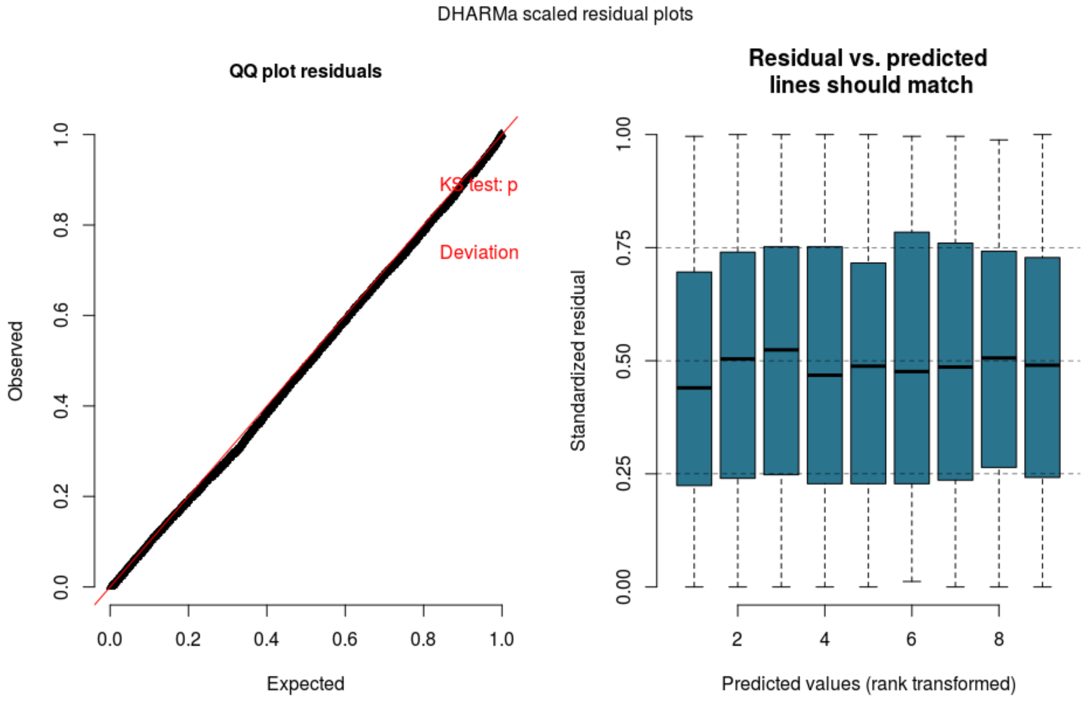

Fig. SI.5 DHARMa diagnostic residual plots of the logistic regression modelling the red deer use of conservation grazing sites in winter.

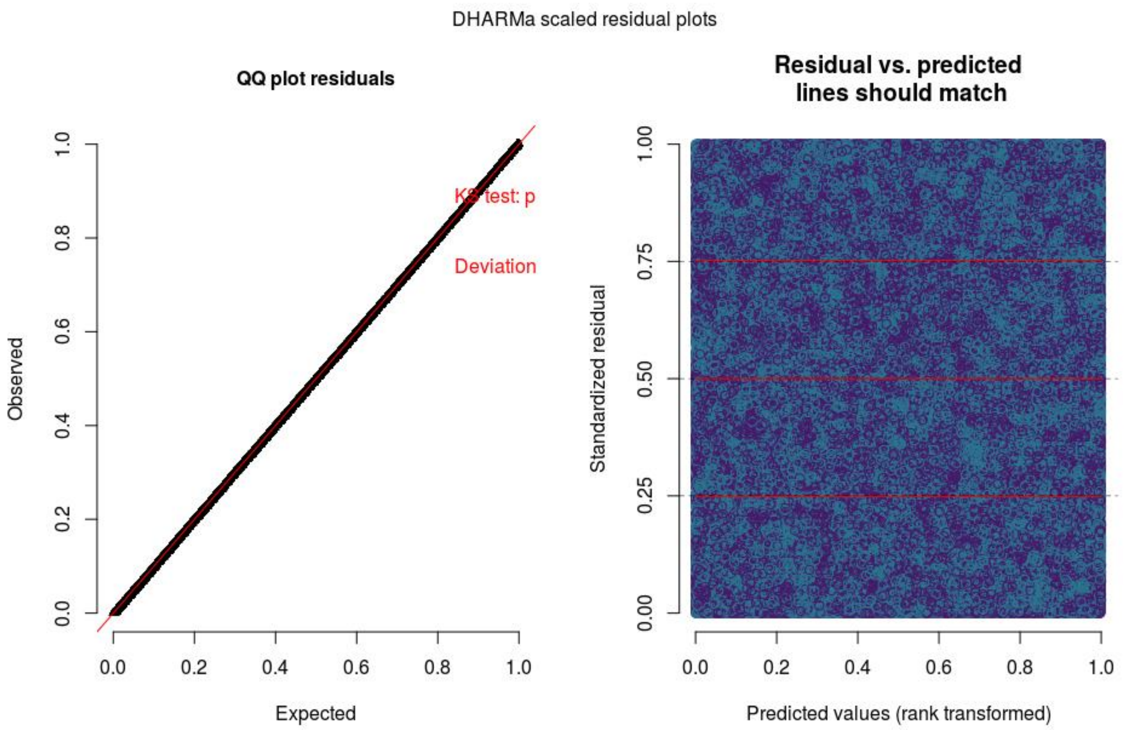

Fig. SI.6 DHARMa diagnostic residual plots of the logistic regression used to derive coefficients of the summer RSF.

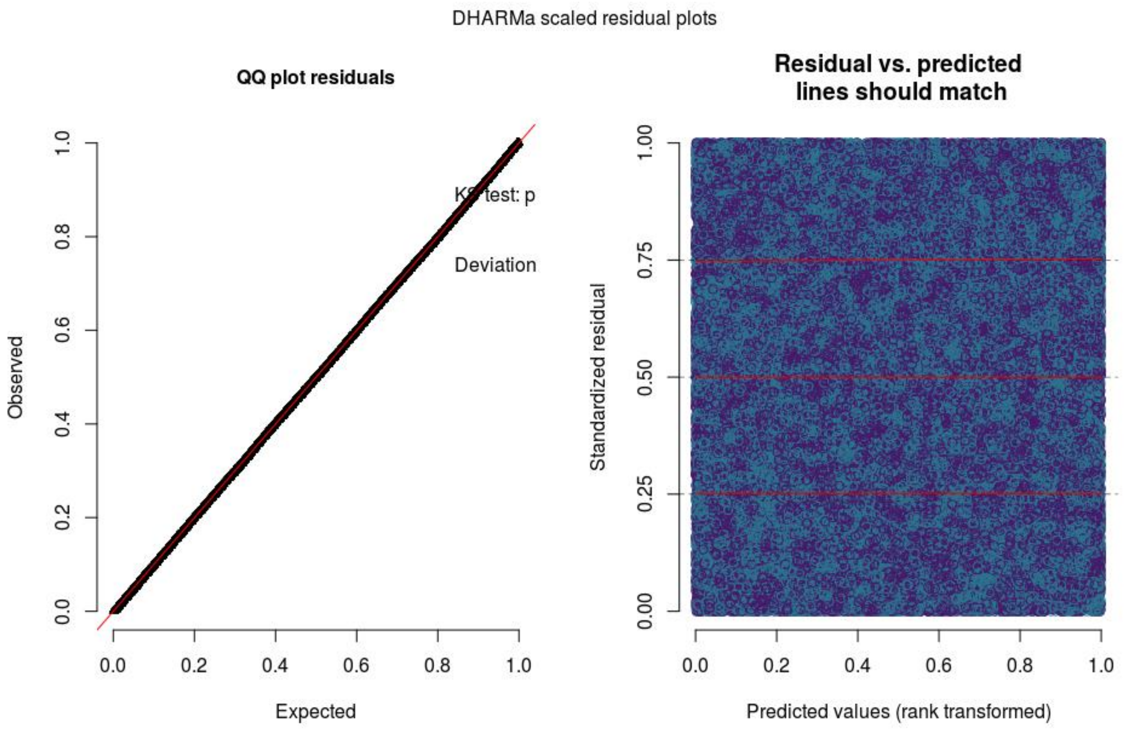

Fig. SI.7 DHARMa diagnostic residual plots of the logistic regression used to derive coefficients of the winter RSF.

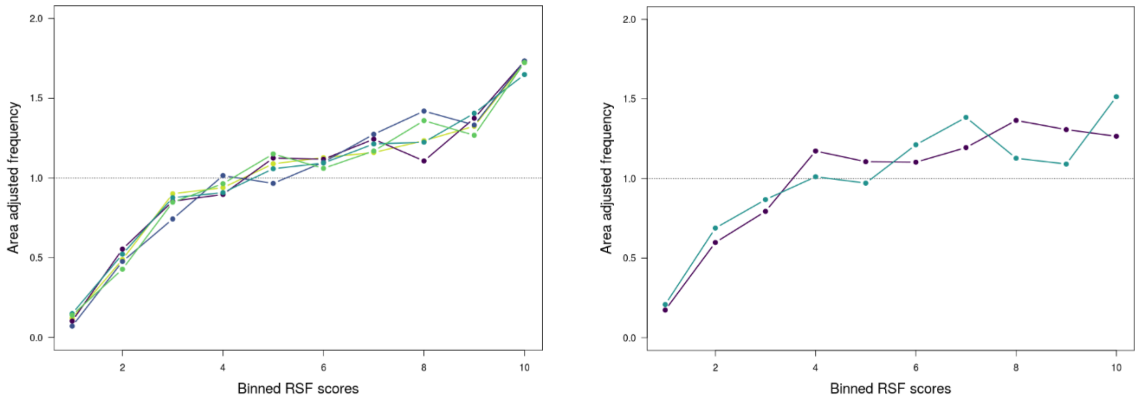

Fig. SI.8 & SI.9 Cross-validation graphs of the summer RSF. **Fig. SI.8 (left)** 5-fold random cross-validation. **Fig. SI.9 (right)** 2-fold blocked cross-validation.

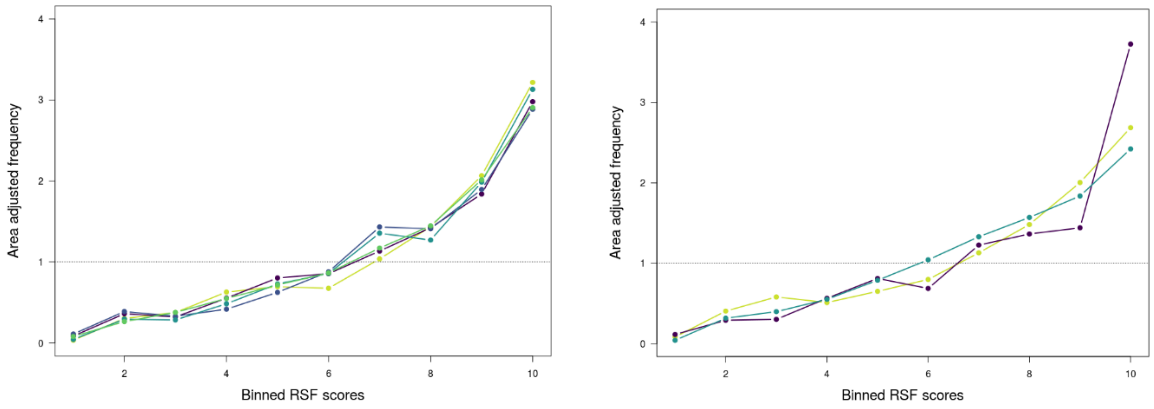

Fig. SI.10 & SI.11 Cross-validation graphs of the winter RSF. **Fig. SI.10 (left)** 5-fold random cross-validation. **Fig. SI.11 (right)** 2-fold blocked cross-validation.

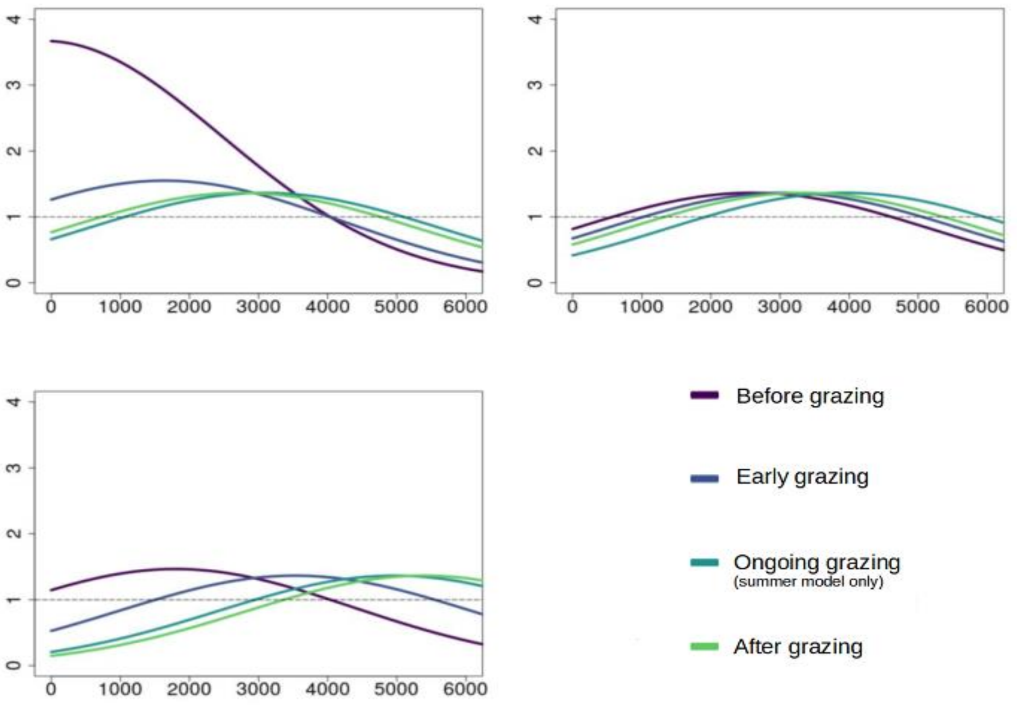

Fig. SI.12 Resource selection for distance to conservation grazing sites during summer for day (**top-left**), night (**top-right**) and twilight (**bottom-left**) with distance (meters) on the x-axis and relative attractiveness (RSF score) on the y-axis.

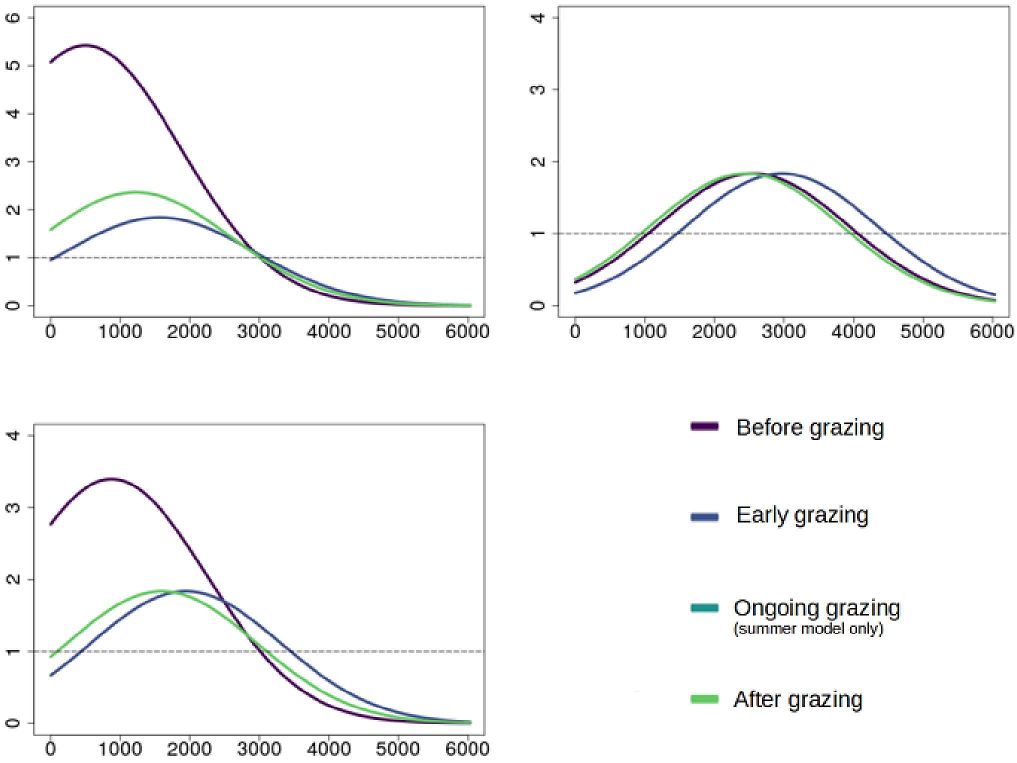

Fig. SI.13 Resource selection for distance to conservation grazing sites during winter for day (**top-left**), night (**top-right**) and twilight (**bottom-left**) with distance (meters) on the x-axis and relative attractiveness (RSF score) on the y-axis.
